# Assessment of Response to Neoadjuvant Chemotherapy in Locally Advanced Breast Carcinoma Using Image-Guided Clip Placement

**DOI:** 10.7759/cureus.47763

**Published:** 2023-10-26

**Authors:** Vikas Pandurangappa, Shivani B Paruthy, Rupi Jamwal, Arun Singh, Sushant Tanwar, Deepak Kumar, Soni Pal, Sajith K Mohan, Anirban Das, Prudhvi Raju TRS

**Affiliations:** 1 Surgery, Vardhman Mahavir Medical College and Safdarjung Hospital, New Delhi, IND; 2 Radiodiagnosis, Vardhman Mahavir Medical College and Safdarjung Hospital, New Delhi, IND

**Keywords:** breast conservation surgery, triple-negative breast carcinoma, pathologic complete response (pcr), complete clinical response, modified radical mastectomy (mrm), recist criteria, neoadjuvant chemotherapy(nact), locally advanced breast-cancer

## Abstract

Background

The present study aims to evaluate the response of locally advanced breast carcinoma (LABC) to neoadjuvant chemotherapy (NACT) using image-guided clip placement based on Response Evaluation Criteria in Solid Tumors (RECIST) 1.1 criteria.

Methods

Thirty-four patients with LABC were included in the study. Consent for three-dimensional titanium clip placement (400/300/200 mm Liga clips) under local anesthesia with USG guidance was obtained. Serial sonographic/X-ray evaluations of tumor bed size were conducted before every cycle of NACT. All data were recorded in millimeters of concentric tumor regression/non-regression. Tumor regression in a concentric or Swiss cheese pattern and non-responders were evaluated. Assessment of the response to NACT was performed using RECIST criteria, dividing it into four categories. Tumor response was confirmed with computerized tomography (CT) conducted before and after the completion of NACT. Patients underwent surgical management, mostly modified radical mastectomy (MRM), as they had locally advanced breast carcinoma. Following MRM, the clips in the specimen guided the original site of the tumor for histopathological evaluation and response to chemotherapy.

Results

Tumor response was classified into four types: complete response (CR), partial response (PR), progressive disease (PD), and stable disease. RECIST 1.1 criteria were elaborated and defined. Data for all patients were entered into an Excel sheet (Microsoft Corporation, Redmond, Washington) to prepare a master chart, and the following observations were made and analyzed using SPSS software. The duration of chemotherapy for the study population ranged from 32 to 206 days, with a mean (±SD) of 111.82 (± 52.64) days and a median (IQR) of 81 (63, 158) days. The mean period between clip insertion and completion of NACT was 111.82 days. The baseline sum diameters and post-NACT diameters of the tumors were 70.50 (±13.60) mm before NACT and 17.75 (±17.20) mm after NACT. Hence, the mean size of the lump was statistically significantly lower after NACT, with a mean difference of 52.75 (p<0.05). The mean rate of reduction in tumor diameter was found to be 74.32% (±23.44%) based on RECIST 1.1 criteria. Pathological response was observed in all patients except for 8.8% of the patients. Clinical complete response was seen in 35.29% of patients, and partial response was observed in 52.92% of the patients based on RECIST 1.1 criteria. The study thus demonstrates the effectiveness of NACT in LABC, with a mean reduction in tumor diameter of 74.32%, assessed with the help of RECIST 1.1 criteria.

Conclusion

NACT for patients with LABC has shown a significant reduction in tumor size. NACT should be the initial mode of management for patients with LABC. RECIST 1.1 criteria are effective and can be used to assess tumor response to NACT. This has aided in the stratification of the response of NACT for further management through systemic therapy (adjuvant chemotherapy) after the surgical excision of the tumor.

## Introduction

Breast carcinoma occurs after puberty but with increasing frequency as age advances, with more disability-adjusted life years (DALYs) by females due to breast cancer globally than any other cancer [1]. Breast carcinoma is a common malignancy among women, accounting for approximately 25% of all cancers [2]. The incidence rate based on race and ethnicity in developed countries is higher [3]. The incidence rate of breast cancer varies in different parts of the world, with a higher incidence among Asian and American women [4].

The incidence of breast cancer is due to various factors, but factors such as genetic factors, environmental factors, significant family history, and lifestyle factors are the most important ones. Factors such as parities, lactation, and exercise reduce the risk of the disease [5]. Screening, chemoprevention, and biological prevention help in reducing mortality in breast cancer patients [6]. Aromatase inhibitors (AIs) and selective estrogen receptor modulators (SERMs) are classes of anti-estrogen drugs used for the chemoprevention of breast cancer [7].

Based on histological characteristics, breast cancer is divided into two types: in situ cancer and invasive breast carcinoma, with the most common invasive cancer being ductal cancer, which accounts for 70-85% of cases. The hormonal status of the patient helps in understanding the etiology of the disease and the response to treatment, with the choice of treatment depending on the expression of individual receptors [8]. Gene expression in breast tumors has been intensively studied and is broadly divided into four subtypes: luminal type A, luminal type B, HER2-positive, and basal cell type [9].

Neoadjuvant chemotherapy (NAC) is the initial treatment modality for operable patients and inoperable breast cancer. This has reduced mortality and has improved surgical options for management by converting inoperable cases to operable ones [10]. Clinical response to NACT was observed in 80-90% of patients [11]. With improvements in the NACT regime and newer chemotherapeutic agents, patients have shown a positive response (approximately 80%), with a dramatic pathological complete response (pCR) observed in 6-32.9% of patients [12,13]. A clinical, radiological, and pathological complete tumor response has complicated surgical excision due to the difficulty in accurately localizing the tumor's site [11,13].

Physical examination, mammography, and sonography are part of the triple assessment used for therapy assessment. Many studies have shown that MRI provides a more accurate determination of the response to chemotherapy than other modalities, and extensive research has been conducted on this [14]. The radiopaque marker is a safe and inexpensive technique used for marking the tumor bed before surgical excision in patients who have shown a complete tumor response after receiving neoadjuvant chemotherapy [12-14].

There is the availability of many breast markers commonly used for tumor localization before NACT. Among the most commonly used are the inexpensive titanium-based metallic clip markers placed using either mammography or sonography. A preliminary report on sonographically guided implantation of metallic clips to permanently localize the tumor bed showed a complete mammographic or sonographic response in 42% of patients [12,15,16]. Radiopaque clip placement in patients who received neoadjuvant chemotherapy demonstrated better local control with reduced local recurrences compared to patients who did not have radiopaque clip placement [11]. The clip was visible with ultrasound in almost 100% of cases. The clip remained visible for a longer period, ranging from 5.9 to a maximum of 7.5 months, which falls within the total chemotherapy period [13].

The Response Evaluation Criteria in Solid Tumors (RECIST) criteria are globally accepted for evaluating the tumor response to NACT and are divided into four categories. RECIST provides a set of rules for image interpretation and assessing tumor reduction [17,18]. Patients who exhibit an adequate response to NACT should predict the pattern of tumor shrinkage before treatment [17].

## Materials and methods

This study was conducted in the Department of General Surgery at Vardhman Mahavir Medical College and Safdarjung Hospital, New Delhi, India. It was designed as an observational cohort study, which took place over a period of 18 months, from December 2020 to May 22, and the study population consisted of patients clinically diagnosed with locally advanced carcinoma breast who were planned for neoadjuvant chemotherapy.

Inclusion and exclusion criteria

The study included patients diagnosed with locally advanced carcinoma breast who were planned for neoadjuvant chemotherapy. Patients with a history of previous radiation treatment, previous breast surgery, or recurrent disease were excluded from the study.

Sample size

The study conducted by Shalaby et al. [19] examined the accuracy of localizing breast malignant masses in patients who underwent neoadjuvant chemotherapy and conservative breast surgery, using clip and wire markers. Based on the findings of this study, a minimum sample size of 24 patients was determined, with a 0.5% margin of error and a 5% level of significance. To further minimize the margin of error, a total sample size of 30 patients was selected.

Calculation

The formula used is: $(\frac{Z&alpha;}{2})^{2}\times P\times \frac{Q}{i\times i}$

where *Z*α is the value of *Z* at a two-sided alpha error of 0.5%, *P* is the proportion, *Q* is (100 - *P*), and *i* is the incidence rate.

n ≥ ((0.000348 × (1 - 0.000348))/(0.005/1.96))^2^ = 23.986 = 24 (approx.)

We have taken a minimum of 30 samples; hence sample size was 30.

Methodology

In this study, a comprehensive assessment of patients was conducted, which included gathering demographic information, medical history, and assessing signs and symptoms. A thorough clinical evaluation was carried out, encompassing a triple assessment involving clinical, radiological, and histopathological assessments. Routine investigations such as chest X-ray, ECG, and routine blood tests were also performed. Pathological assessment was done through FNAC (fine needle aspiration cytology) and core needle biopsy, including the evaluation of ER, PR, and Her2 neu status, along with identifying the type of breast cancer.

**Figure 1:**
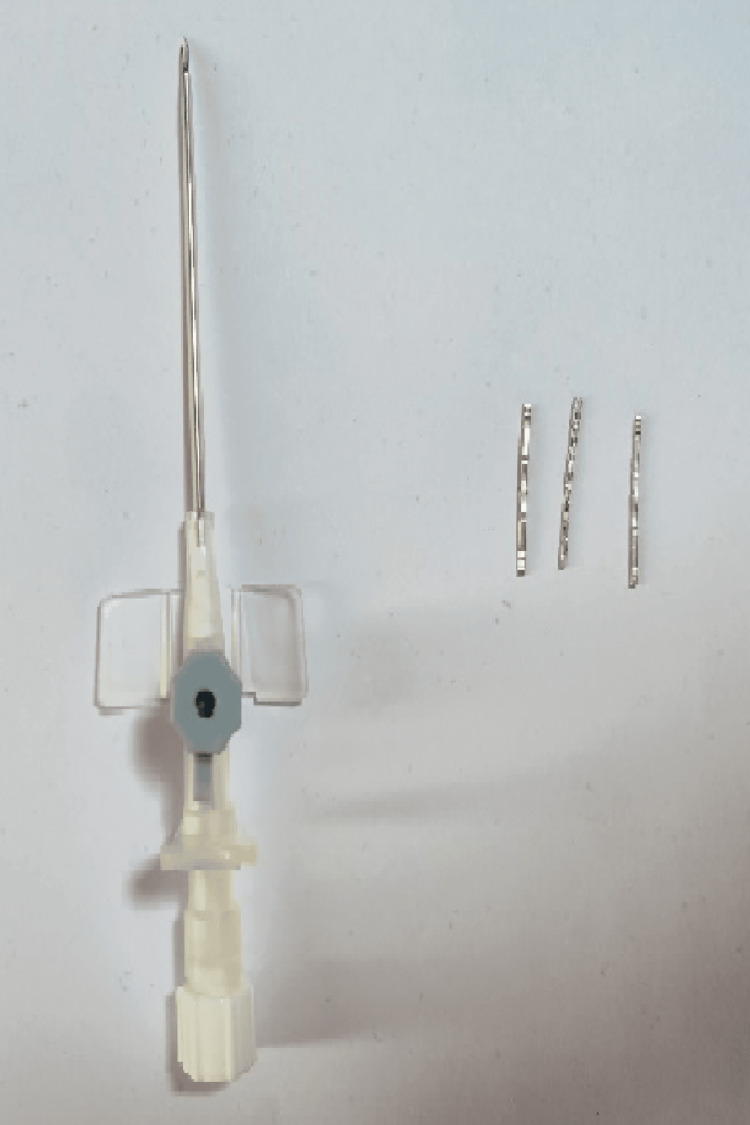
A 16-gauge cannula needle with a titanium clip (300 mm Liga clips).

Patients who met the inclusion criteria and were planned for neoadjuvant chemotherapy based on metastatic workup were included in the study. Consent was obtained for the placement of three-dimensional titanium clips (400/300/200 mm Liga clips) under local anesthesia with USG guidance. A sonographic assessment of tumor size and margins was conducted. A surgical tray was prepared, including 16-gauge cannula needles (4), antiseptic draping material, Liga clips of various sizes, local anesthesia, syringe, and a needle with a sterile cover for the ultrasound probe to facilitate the placement of the clips (Figure 1).

**Figure 2:**
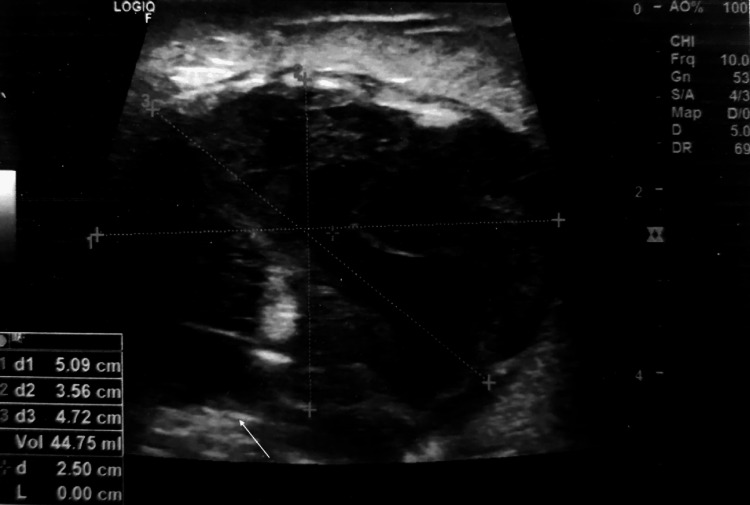
Sonographic marking of a tumor with a surgical clip seen in situ (white arrow).

With aseptic precautions, a plastic cannula with stylet was placed at tumor margins under USG guidance. A plastic cannula was disengaged with the distal end of the hub. The surgical clip was straightened and mounted on the plastic cannula. The stylet was pushed to place the clip in an appropriate position and doubly checked by USG guidance (Figure 2).

**Figure 3:**
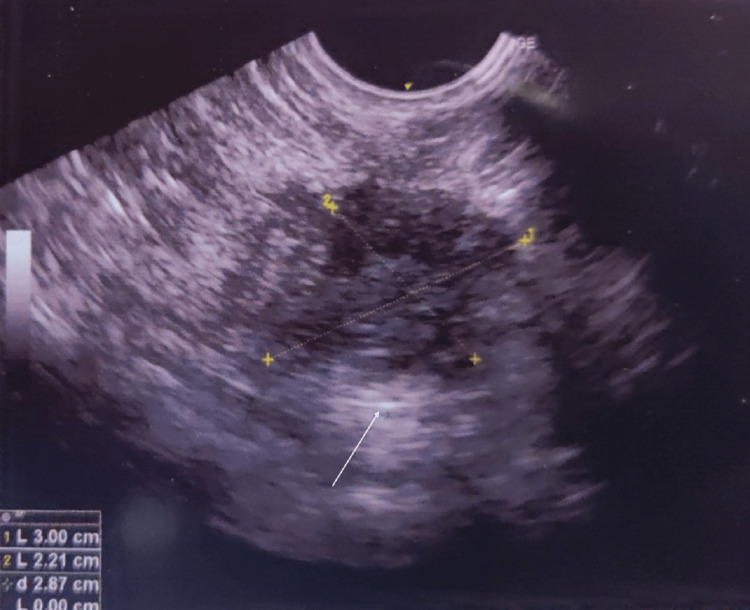
A radiopaque clip noted at the tumor base. The surgical clip (white arrow) is noted over the tumor bed after two cycles of chemotherapy.

**Figure 4:**
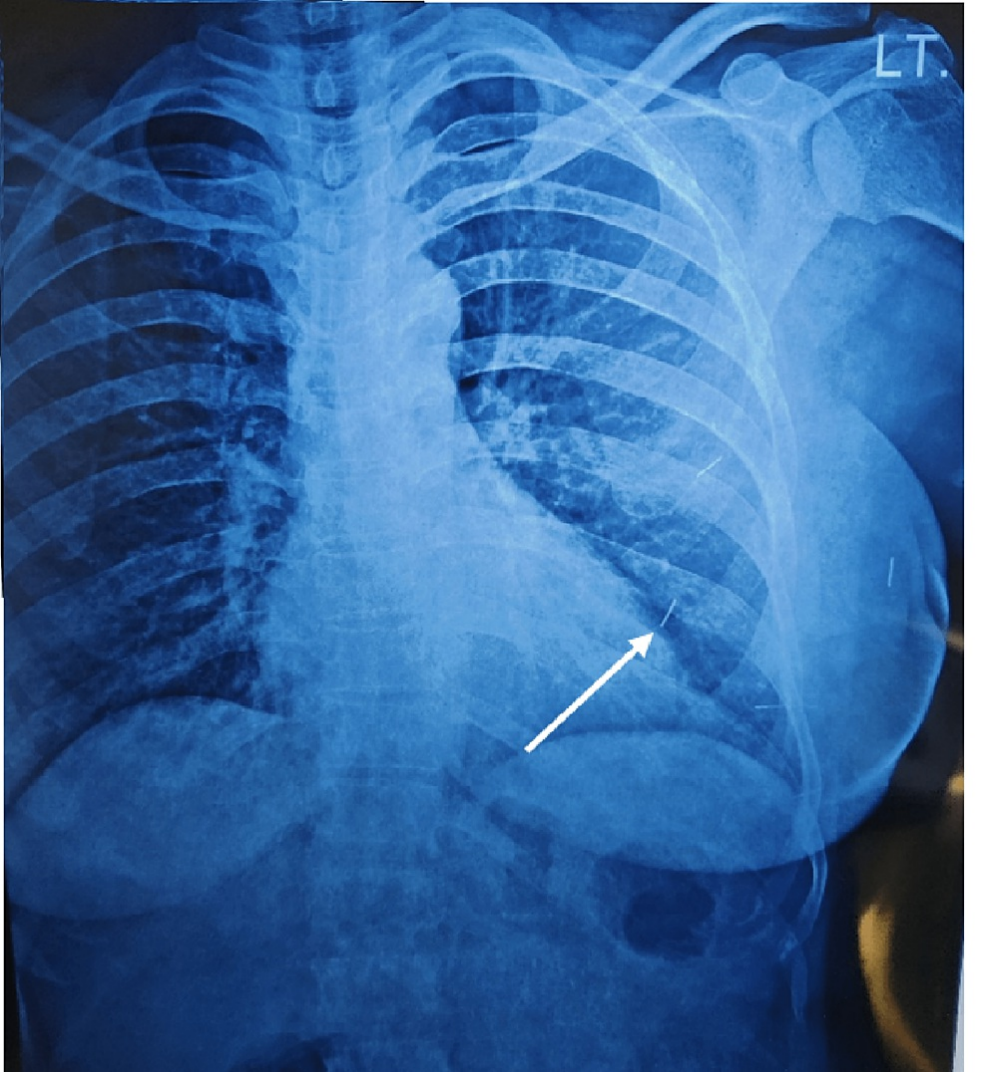
Chest X-ray showing inserted surgical clips (white arrow) used for tumor localization.

Four such passes were made for all four corners and one pass was made for the depth of the tumor and one for the superficial extent (Figures 3, 4).

Bigger tumors required additional passes and the placement of extra clips. The tumor bed was localized, and a clip was placed in the same manner. Serial sonographic/X-ray evaluations of the tumor bed size were conducted before every cycle of neo-adjuvant chemotherapy. The assessment of actual tumor regression in responders to neo-adjuvant chemotherapy was guided by the surgical clips in situ.

All data were recorded in millimeters of concentric tumor regression or non-regression. Tumor regression in either concentric or "Swiss cheese" patterns and non-responders were evaluated.

Assessment of response to NACT was performed using RECIST criteria, dividing it into four categories. Tumor response was confirmed with computerized tomography (CT) scans taken before and after completion of NACT. Patients were subjected to surgical management, mostly modified radical mastectomy (MRM), as patients in the study group had locally advanced breast carcinoma. Following MRM, the clips in the specimen guided the original site of the tumor for histopathological evaluation and response to chemotherapy.

Statistical analysis

Categorical variables were presented in number and percentage and continuous variables were presented as mean ± SD and median. A diagnostic test was used to calculate sensitivity, specificity, PPV, and NPV. Inter-rater kappa agreement was used to find out the strength of agreement between the predicted response and the actual response. A p-value of <0.05 was considered statistically significant. The data was entered in an MS Excel (Microsoft Corporation, Redmond, Washington) spreadsheet and analysis was done using IBM SPSS Statistics for Windows, Version 21 (Released 2012; IBM Corp., Armonk, New York). The type of the study was an observational cohort study.

## Results

A total of 34 patients were included in the study. The age of the study population ranged from 32 to 70 years, with a mean (± SD) of 48.79 (± 10.87) years and a median (interquartile range (IQR)) of 47 (40, 58) years. Of these patients, 55.9% were younger than 50 years and 44.1% were 50 years or older. In terms of symptoms, 38.2% of the patients had a lump, 50% had a lump with pain, and 11.8% had a lump with pain and nipple discharge. The median (IQR) lump size was found to be 6×5 (5×5, 7×6) cm.

The age at menarche of the study population ranged from 12 to 16 years, with a mean (± SD) of 13.29 (± 1.12) years and a median (IQR) of 13 (12, 14) years. Specifically, 58.8% of the patients experienced menarche at ages 12 to 13, and 41.2% at ages 14 to 15.

Regarding menopausal status, 55.9% of the patients were pre-menopause and 44.1% were post-menopause. For those in post-menopause, the age at menopause ranged from 45 to 54 years, with a mean (± SD) of 50.20 (± 2.48) years and a median (IQR) of 50 (48, 52) years. The number of pregnancies was one for 20.6% of the patients, two for 58.8%, three for 17.6%, and four for 2.9%.

According to the BIRADS score, 2.9% were graded as 4C, indicating high suspicion for malignancy; 67.6% were graded as 5, highly suggestive of malignancy; and 29.4% were graded as 6, signifying known biopsy-proven malignancy. The surrogate molecular classes were as follows: 29.4% were HER2/neu-enriched, 2.9% were luminal A, 44.1% were luminal B, and 23.5% were triple-negative.

The duration of clip to NAC 1 ranged from 1 to 18 days, with a mean (± SD) of 5.71 (± 3.74) days and a median (IQR) of 5 (4, 6) days. The duration of chemotherapy ranged from 32 to 206 days, with a mean (± SD) of 111.82 (± 52.64) days and a median (IQR) of 81 (63, 158) days (Table 1).

**Table 1:** Duration of clip to NAC 1 and duration of chemo (N = 34). NAC: neoadjuvant chemotherapy, IQR: interquartile range.

Variables	Mean ± SD	Median (IQR)	Minimum	Maximum
Duration of clip to NAC 1 (days)	5.71±3.74	5(4,6)	1	18
Duration of chemo (days)	111.82±52.64	81(63,158)	32	206

**Figure 5:**
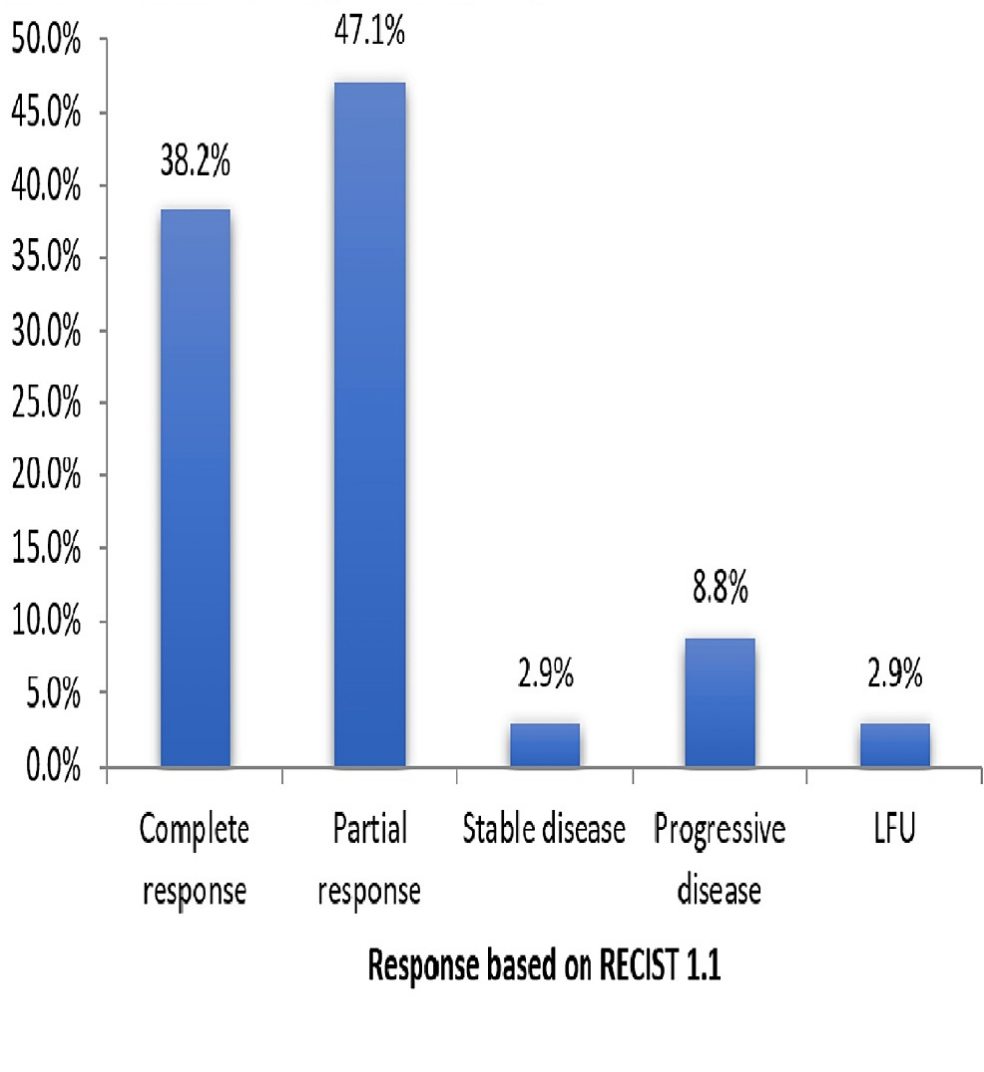
Response based on RECIST 1.1 in the study population (N = 34). LFU: lost to follow-up.

According to RECIST 1.1, there was a complete response by 38.2% of the patients, a partial response by 47.1% of the patients, a stable disease for 2.9% of the patients, and a progressive disease for 8.8% of the patients (Figure 5).

One patient (2.9%) was lost to follow-up (Table 2).

**Table 2:** Response based on RECIST 1.1 in the study population (N=34). LFU: lost to follow-up.

Response based on RECIST 1.1	Frequency	Percentage
Complete response	13	38.2%
Partial response	16	47.1%
Stable disease	1	2.9%
Progressive disease	3	8.8%
LFU	1	2.9%

The post-surgery plan was adjuvant chemotherapy for 33 patients, hormonal therapy for 19 patients, and follow-up for 18 patients. The mean (±SD) size of the lump before NACT was 70.50 (±13.60) before NACT, whereas it was 17.75 (±17.20) mm after NACT. Hence the mean size of the lump was statistically significantly lower after NACT as compared to before NACT with a mean difference of 52.75 (p < 0.05) (Table 3). The mean (±SD) of the percentage of response in the study population was found to be 74.32 (±23.44)%.

**Table 3:** Comparison of size of lump before and after NACT. NACT: neoadjuvant chemotherapy.

Time	N	Mean ± SD size of lump	Mean difference	P-value
Before NACT	33	70.50±13.60	52.75 (45.18-60.33)	<0.001
After NACT	33	17.75±17.20		

## Discussion

This observational cohort study was conducted in the Department of General Surgery at Vardhman Mahavir Medical College and Safdarjung Hospital, New Delhi. A total of 34 patients with locally advanced breast carcinoma were selected based on a triple assessment, which included clinical, radiological, and pathological evaluations. Patients who were eligible for NACT were chosen. Between December 2020 and May 2022, these patients received NACT. Prior to administering neo-adjuvant chemotherapy, the tumor bed and margins were marked using sonographically guided surgical clip placement. All patients underwent neo-adjuvant chemotherapy, and the tumor response was assessed using RECIST 1.1 criteria. This assessment divided tumor response into four categories: complete response (CR), partial response (PR), progressive disease (PD), and stable disease (SD).

In the study by Shalaby et al. [19], tumor response was assessed by comparing image findings after two cycles of chemotherapy, facilitated by clip placement. Wire localization was performed on radiopaque clips at the end of NACT in patients with non-palpable lesions, providing an accurate and straightforward approach for the surgeon to the tumor bed. Our study follows a similar methodology.

The overall stage of the disease was determined by considering clinical tumor size, lymph node status, and distant metastasis. In our study, 8.8% were in clinical stage 3A, 67.6% in stage 3B, and 23.5% in stage 4. According to the BIRADS score, 67.6% were graded as 5, indicating they were highly suggestive of malignancy, while 29.4% were graded as 6, signifying known biopsy-proven malignancy. These results align closely with the study by Shalaby et al. [19], where all 20 patients studied had pathologically proven malignancies (i.e., BI-RADS VI) and were diagnosed with locally advanced breast carcinoma (stages IIB, IIIA, and IIIB).

Immunohistochemical evaluations of receptors were conducted on all 34 breast tumor specimens. The surrogate molecular class was HER2/neu-enriched for 29.4% of the patients, luminal A for 2.9%, luminal B for 44.1%, and triple-negative for 23.5%. All the lesions were invasive ductal carcinoma of non-specific type. A related study by Minella et al. [16] included 52 patients, all with non-specific type tumors. Of these, 25% were categorized as triple-negative breast cancers (TNBC), 50% as HER2-positive tumors, and the remaining 25% as ER-positive/HER2-negative tumors.

Clip localization was performed on all patients in our study group (n = 35). No complications related to clip placement were observed during the study period, and there was no evidence of clip migration during post-procedural follow-up, post-NACT, or in surgical specimens. Similar outcomes were reported in the study by Youn et al. [20], which also found no evidence of clip migration or other complications related to clip insertion.

The time interval between clip insertion and the first cycle of NACT in our study population ranged from 1 to 18 days, with a mean (±SD) of 5.71 (±3.74) days and a median (IQR) of 5 (4, 6) days. The duration of chemotherapy ranged from 32 to 206 days, with a mean (± SD) of 111.82 (± 52.64) days and a median (IQR) of 81 (63, 158) days. The mean period between clip insertion and completion of NACT was 111.82 days. According to a study by Youn et al., the time intervals between clip insertion, post-chemotherapy cycles, and surgery were similar, with mean periods of 47.1±14.7 days and 128.6±34.4 days, respectively [20].

Surgery was scheduled approximately 2 to 4 weeks after the completion of NACT, in line with current guidelines. Using CT, the baseline sum of tumor diameters was 70.50 (±13.60) mm before NACT and reduced to 17.75 (±17.20) mm after NACT. This represented a statistically significant reduction in tumor size, with a mean difference of 52.75 mm (p < 0.05). 

The rate of reduction in tumor diameter was found to be 74.32 (±23.44)%, based on RECIST 1.1 criteria. Treatment response, as measured by CT and evaluated through RECIST 1.1, showed CR in 12 patients (35.28%), PR in 18 patients (52.92%), stable disease in 2 patients (5.8%), and progressive disease in 1 patient (2.94%). One patient (2.9%) was lost to follow-up. 

A similar study by Kitajima et al. showed a comparable rate of tumor diameter reduction of 69.9±31.6% [21]. These findings align closely with the results of our study, indicating a robust and consistent approach to assessing tumor response to NACT in patients with locally advanced breast carcinoma.

In this study, the pathological response was observed in all patients except for 5.8%. A complete response was seen in 35.29% of patients, and a partial response in 52.92% based on RECIST 1.1 criteria. A study by Oh et al. showed a similar pathological response in all but 3.8% of the patients [11].

In this study, radiological rCR was found in 35.29% of the patients, all of whom had surgical clip insertion done. Clips were visible sonographically in 100% of the patients after NACT. All patients underwent MRM based on patient choice. These results were similar to the study by Minella et al., which showed that rCR was found in 53.7% of the patients. Of these, clip insertion was done in 65%, and clips were visible in 91% of the patients after NACT. Following NACT, 26 patients underwent breast conservative surgery, while the remainder underwent mastectomy based on patient choice [16].

This study showed a cCR and an rCR in 35.29% of cases with the surrogate molecular class of triple-negative or basal cell variant. This is similar to the study by Xia et al., which showed a pCR rate of 35% among 36,480 triple-negative patients receiving neoadjuvant chemotherapy [22].

Strong relationships exist between the response of locally advanced breast carcinoma to primary systemic therapy (neoadjuvant chemotherapy) and oncological outcomes. Patients are stratified based on pathological response [21]. This stratification aids in studying new systemic therapies and new combinations of treatment modalities.

Stratification of the response to neoadjuvant chemotherapy was done based on the latest NCCN guidelines. It is broadly divided into two groups: tumors with pCR and tumors with partial response, stable disease, or progressive disease.

After stratification into various groups, patients were referred to the Department of Medical Oncology for assessment and further management, including adjuvant chemotherapy (Tables 4, 5).

**Table 4:** Adjuvant chemotherapy for advanced breast carcinoma in tumors with pCR response.

Hormonal status (ER, PR status)	HER2/neu status	
	Positive	Negative
Positive	Endocrine therapy + 1 year of HER-targeted therapy with trastuzumab/pertuzumab	Adjuvant endocrine therapy + targeted therapy for BRCA 1/2 germline mutation
Negative	1 year of HER2-targeted therapy with trastuzumab/pertuzumab.	Adjuvant pembrolizumab

**Table 5:** Adjuvant chemotherapy for advanced breast carcinoma in tumors with partial response, stable disease, or progressive disease.

Hormonal status (ER, PR status)	HER2/neu status	
	Positive	Negative
Positive	Endocrine therapy + 1 year of HER-targeted therapy with trastuzumab/pertuzumab	Adjuvant endocrine therapy + targeted therapy for germline BRCA1/2 mutation
Negative	Ado-transtuzumab emtansine for 14 cycles or 1 year of HER2-targeted therapy with trastuzumab/pertuzumab	Adjuvant capecitabine for 6-8 cycles or adjuvant olaparib for 1 year in case of germline BRCA1/2 mutation or adjuvant pembrolizumab

## Conclusions

The duration of NACT in the study population was adequate, ranging from three to six months. The impact of NACT on tumor size was assessed using standard RECIST 1.1 criteria, which showed a statistically significant reduction in tumor diameter. Only approximately 8% of the study population showed no response to NACT, while about 35% experienced a complete response with no residual tumor noted. Therefore, the study demonstrates the effectiveness of NACT in locally advanced breast carcinoma, with a mean reduction in tumor diameter observed to be 74%, as assessed by RECIST 1.1 criteria. This implies that NACT should be the initial mode of management for patients with locally advanced breast carcinoma. This has aided in the stratification of the NACT response for further systemic therapy following surgical excision of the tumor, thereby improving the oncological outcomes for patients.
